# Dopamine-induced tyrosine phosphorylation of NR2B (Tyr1472) is essential for ERK1/2 activation and processing of novel taste information

**DOI:** 10.3389/fnmol.2014.00066

**Published:** 2014-07-18

**Authors:** Orit David, Iliana Barrera, Adaikkan Chinnakkaruppan, Hanoch Kaphzan, Takanobu Nakazawa, Tadashi Yamamoto, Kobi Rosenblum

**Affiliations:** ^1^Sagol Department of Neurobiology, University of HaifaHaifa, Israel; ^2^Division of Oncology, Institute of Medical Science, University of TokyoTokyo, Japan; ^3^Center for Gene Manipulation in the Brain, University of HaifaHaifa, Israel

**Keywords:** dopamine, acetylcholine, extracellular signal-regulated kinase (ERK), NR2B, memory, cortex, hippocampus

## Abstract

Understanding the heterosynaptic interaction between glutamatergic and neuromodulatory synapses is highly important for revealing brain function in health and disease. For instance, the interaction between dopamine and glutamate neurotransmission is vital for memory and synaptic plasticity consolidation, and it is known to converge on extracellular signal-regulated kinase (ERK)-MAPK signaling in neurons. Previous studies suggest that dopamine induces *N*-methyl-D-aspartate (NMDA) receptor phosphorylation at the NR2B Y1472 subunit, influencing receptor internalization at the synaptic plasma membrane. However, it is unclear whether this phosphorylation is upstream to and/or necessary for ERK1/2 activation, which is known to be crucial for synaptic plasticity and memory consolidation. Here, we tested the hypothesis that tyrosine phosphorylation of NR2B at Y1472 is correlated with ERK1/2 activation by dopamine and necessary for it as well. We find that dopamine receptor D1, but not D2, activates ERK1/2 and leads to NR2BY1472 phosphorylation in the mature hippocampus and cortex. Moreover, our results indicate that NR2B Y1472 phosphorylation is necessary for ERK1/2 activation. Importantly, application of dopamine or the D1 receptor agonist SKF38393 to hippocampal slices from NR2B F1472 mutant mice did not result in ERK1/2 activation, suggesting this site is not only correlated with ERK1/2 activation by dopamine stimulation, but also necessary for it. In addition, NR2B F1472 mice show impairment in learning of attenuation of taste neophobia but not associative taste learning. Our study shows that the dopaminergic and glutamatergic transmission converge on the NMDA receptor itself, at the Y1472 site of the NR2B subunit, and that this convergence is essential for ERK1/2 activation in the mature brain and for processing new sensory information in the cortex.

## INTRODUCTION

Memory consolidation is enabled, at least in part, by heterosynaptic modulation of the glutamatergic input by neuromodulatory synapses in different brain areas (Bailey et al., 2000). One such interaction is the heterosynaptic modulation between the ionotropic glutamate receptor, *N*-methyl-D-aspartate (NMDA) receptor and metabotropic dopamine receptors. Interaction between glutamatergic and dopaminergic synapses in the mature hippocampus is important for modulation of long-term potentiation (LTP) and long-term memory in the CA1 region of the hippocampus (Frey et al., 1990, 1991; Matthies et al., 1997; Bailey et al., 2000). Blocking this interaction prevents or attenuates LTP (Frey et al., 1990, 1991; Huang and Kandel, 1995; Riedel et al., 2003). We have previously shown that dopamine and NMDA induce ERK1/2 activation in the mature hippocampus, and that the convergence of these two signaling pathways takes place on the NMDA receptor itself (Kaphzan et al., 2006, 2007). However, the detailed molecular mechanism underlying this convergence has been largely unexplored.

From a biochemical perspective, D1- and D2-type dopamine receptors activate MAPK cascades, such as ERK, p38, and JNK in neuronal as well as in non-neural cells (Yan et al., 1999; Valjent et al., 2000; Guerrero et al., 2002; Lee et al., 2006). Phosphorylation of the NR2B subunit of the NMDA receptor at Y1472 located at the intracellular c-terminal end of the protein is mediated by the Src-protein family of kinases (SFK; Grosshans et al., 2002). In addition, Fyn protein tyrosine kinase is required for NMDA receptor tyrosine phosphorylation (Dunah and Standaert, 2001; Dunah et al., 2004).

The NMDA receptor is involved in various aspects of synaptic plasticity induction and memory acquisition in different brain structures underlying different learning forms and potentiation paradigms (Bliss and Collingridge, 1993; Bear and Abraham, 1996; Kemp and Bashir, 2001). Phosphorylation of the NR2B subunit of the NMDA receptor at Y1472 is involved in maintenance of neuropathic pain (Abe et al., 2005). This phosphorylation is also required for both synaptic plasticity (Nakazawa et al., 2002, 2006) and learning paradigms such as fear conditioning (Nakazawa et al., 2006).

Numerous studies have shown the importance of the dopaminergic system, NR2B Y1472, and the ERK-MAPK pathway in novel taste learning and conditioned taste aversion (CTA, Berman et al., 2000; Swank, 2000; Belelovsky et al., 2005; Cannon et al., 2005; Elkobi et al., 2008; Barki-Harrington et al., 2009; Guzman-Ramos et al., 2010). However, it is unclear whether these molecular pathways are all linked together in this type of learning.

In the present study, we investigated the mechanisms underlying dopaminergic and glutamatergic synergistic activation of ERK-MAPK pathway via convergence on the NMDA receptor itself, in the mature hippocampus and cortex. We show that dopamine D1 receptor activation induces the Y1472 phosphorylation of NR2B via the Src protein kinase, and that this interaction is essential for cortical-dependent novel taste learning.

## MATERIALS AND METHODS

### ANIMALS

Male Sprague–Dawley rats (∼60 days old, 250–300 g), and C57BL/6 mice (20–22 g) were procured from Harlan (Harlan, Jerusalem, Israel). NR2B F1472 mutant mice lacking the Y1472 phosphorylation site by substituting Tyr-1472 (Y) with Phe-1472 (F) were given to us by Prof. Nakazawa and Prof. Yamamoto (Nakazawa et al., 2006). Animals were provided *ad libitum* with standard food and water and were maintained on a 12/12 h light/dark cycle. All experiments were approved by the Institutional Animal Care and Use Committee of the University of Haifa, and adequate measures were taken in order to minimize pain, in accordance with the guidelines laid down by the European Union and United States NIH, regarding the care and use of animals in experiments.

### PHARMACOLOGY

For pharmacological manipulations: dopamine (20 μM,10 min) was co-applied with the antioxidant ascorbic acid (1 mM); carbachol 2-[(aminocarbonyl)oxy]-*N,N,N*-trimethylethanaminium chloride); D(-)-APV (D-2-amino-5-phosphonovaleric acid (40 μM), dopamine receptor D1 agonist R-(+)-SKF38393 (20 μM, 10 min); D1 antagonist R-(+)-SCH-23390 (R(+)-7-chloro-8-hydroxy-3-methyl-1-phenyl-2,3,4,5-tetrahydro-1*H*-3-benzazepine hydrochloride (40 μM); D2 agonist (-)-quinpirole hydrochloride (20 μM, 10 min); and D2 antagonist eticlopride (3-chloro-5-ethyl-*N*-{[(2*S*)-1-ethylpyrrolidin-2-yl] methyl}-6-hydroxy-2-methoxybenzamide (60 μM), were purchased from Sigma Aldrich. Src-family kinase inhibitor, PP2 (4-amino-5-(4-chlorophenyl)-7-(*t*-butyl)pyrazolo[3,4-*d*]pyrimidine (10 μM) was purchased from Calbiochem. All compounds were dissolved in artificial cerebro-spinal fluid (ACSF) on the day of the experiment.

### HIPPOCAMPAL AND INSULAR CORTEX SLICE PREPARATION

After decapitation, the brain was immediately immersed in cold (4°C) carboxygenated (95% O_2_, 5% CO_2_; ACSF, comprising 124 mM NaCl, 5 mM KCl, 1.2 mM MgSO_4_, 1.2 mM NaH_2_PO_4_, 26 mM NaHCO_3_, 10 mM D-glucose, and 2.4 mM CaCl_2_), and after approximately 2 min, both hippocampi or insular cortex were dissected out in a plate filled with cold (4°C) ACSF on ice. The hippocampi or insular cortex were then put on a cooled stand of a McIlwain tissue chopper TC752 (Campden Instruments Ltd, UK), cut into 400 μm slices, and then put back into a chamber filled with carboxygenated cold (4°C) ACSF (Kaphzan et al., 2006, 2007).

The slices were kept in six holding chambers designed by Scientific Systems Design Company (Ontario, Canada) heated to 32°C and kept for 3 h before any pharmacological intervention. Each chamber contained four slices. Within each experiment, two chambers were used as a positive control for the quality of the slices. The remaining four chambers were the experiment chambers. 10 min. after the pharmacological manipulation, slices were removed from the pharmacological chamber and snap-frozen on dry ice. After freezing, the slices were homogenized in 200 μl of SDS sample buffer, comprising: 1 ml glycerol, 2 ml of 10% SDS, 1.2 ml of 0.5% Tris-HCl (pH 6.8), 4.8 ml of DDW, and 0.5 ml of β-mercaptoethanol. Each specimen comprised two hippocampal/insular cortex slices combined (i.e., 2 slices are *n* = 1).

### DRUG ADMINISTRATION

SKF38393 was dissolved in saline (0.9% NaCl) and administered intraperitoneally (5 mg/kg) to C57BL/6 and NR2B F1472 mice. Mice were sacrificed 15 min later and tissue was collected. For incidental taste learning experiment, SCH23390 was dissolved in saline (0.9%) and administered intraperitoneally (i.p., 0.05 mg/kg) to C57BL/6 mice 30 min prior to the novel taste exposure.

### BIOCHEMISTRY

#### Collection of tissue samples

Fifteen minutes following SKF38393 injection (5 mg/kg, i.p.) or 20 min following novel taste exposure, animals were decapitated and the hippocampus and insular cortex were collected and frozen in liquid nitrogen and stored in -80°C. The obtained tissues were homogenized in a glass-teflon homogenizer in a lysis buffer containing 10 mM HEPES, pH 7.4, 2 mM EDTA, 2 mM EGTA, 0.5 mM DTT, 1% phosphate inhibitor mixture (Sigma), and 1% protease inhibitor mixture (Sigma). Protein quantification was made with BCA Protein Assay Kit (GE Healthcare). Appropriate volumes of 2XSDS sample buffer (10% glycerol, 5% β-mercaptoethanol, 4% SDS, 120 mM Tris-HCl, pH 6.8) were added to the homogenates, and samples were boiled for 5 min and stored at -20°C.

#### Western blotting

Samples in SDS sample buffer were subjected to SDS-PAGE (7.5–10%) and Western blot analysis. Lanes were loaded with equal amount of protein. Following transfer to a nitrocellulose membrane (0.45 μm, Whatman), bands were visualized with Ponceau staining (Bio-Rad). Membranes were blocked in 3–5% BSA (depending on the primary antibody) for 1 h at room temperature, before being incubated overnight at 4°C with the primary antibodies: p44/42 MAP kinase (1:1000) and (Phospho-P44/42 MAP Kinase-(Thr202/Tyr204, 1:1000, Cell Signaling); Src (1:500, Upstate Biotechnology); NR2B antibody (1:500), β-actin (1:3000, Santa-Cruz Biotechnology); Phospho-(Tyr1472)-NR2B (1:500, Embel); and Phospho-(Tyr418)-Src (1:500, Embel).

Following three washing steps with Tris-buffered saline (140 mM NaCl, 20 mM Tris, pH 7.6) plus 0.1% Tween 20 (TBST), membranes were incubated for 1 h at room temperature with secondary HRP-linked antibodies: goat-anti-Rabbit (IgG) HRP conjugated; Goat anti-mouse (IgG) HRP conjugated; and rabbit anti-goat (IgG) HRP conjugated (1:10,000, Jackson ImmunoResearch). Immunodetection was accomplished with the Enhanced Chemiluminescence EZ ECL kit (Biological Industries). Quantification of immunoblots was performed with a CCD camera and Quantity One software (Bio-Rad). Each sample was measured relative to the background. Phosphorylation levels were calculated as the ratio of phosphorylated protein and total amount of protein.

### BEHAVIOR

#### Conditioned taste aversion

***CTA acquisition and extinction.*** Conditioned taste aversion was performed as described previously (Rosenblum et al., 1993; Barki-Harrington et al., 2009). Saccharin (0.5% w/v, sodium salt) was used as the unfamiliar taste in training, i.e., the conditioned stimulus (CS), and i.p. injection of LiCl (2% of the body weight) as the malaise-inducing agent, i.e., unconditioned stimulus (US). The mice were trained for 3 days to drink water once a day during a 20-min period from two pipettes, each containing 5 ml of water. On the conditioning (i.e., fourth) day, they were allowed to drink the saccharin solution containing 0.5% sodium saccharin, instead of water, from similar pipettes during a 20-min period, and 40 min later they were injected with 0.14M LiCl solution at 2% body weight. Two days after conditioning, the mice were tested in a multiple-choice test involving two pipettes with water and two with saccharin. The behavioral data are presented in terms of an aversion index, expressed as [ml water/(ml water + ml saccharin)] × 100 consumed in the test. The greater the preference for water over the novel taste, the higher the aversion index and, therefore, better memory.

#### Incidental taste learning

Mice were separated into individual housing cages and underwent 3-day water-restriction training, in which they were offered 10 ml of water from two pipettes, each containing 5 ml once a day for 20 min. During days 4–8, the mice were given a multiple-choice test comprising two pipettes with water and two with saccharin, during a 20-min period (Rosenblum et al., 1993). The behavioral data are presented in terms of aversion index, defined as [ml water/(ml water + ml saccharin)] consumed in the test.

### STATISTICS

Results are expressed as means ± SEM. All data complied with the normality distribution, determined by the Shapiro–Wilk test. The significance of differences was evaluated using one-way ANOVA followed by Tukey’s *post-hoc* test. For behavioral studies Student’s *t*-test, repeated-measures ANOVA and two-way ANOVA were performed. Significant levels were noted as follows: ^∗^*p* < 0.05, ^∗∗^*p* < 0.01, ^∗∗∗^*p* < 0.001.

## RESULTS

### DOPAMINE D1 BUT NOT D2 RECEPTORS ACTIVATE ERK1/2 IN THE MATURE HIPPOCAMPUS AND CORTEX

We have previously shown that dopamine and NMDA converge to activate ERK1/2 and that this convergence is dependent on the NMDA receptor itself (Kaphzan et al., 2006, 2007). In the present study, we determined which dopamine receptor is involved in this interaction in the mature hippocampus and cortex. Acute rat hippocampal slices were incubated with 20 μM dopamine. As expected, dopamine induced ERK1/2 activation (1.26 ± 0.03, *p* < 0.001, *n* = 24; Figure S1A) and blocking both the D1 receptor (SCH23390, 40 μM) and the D2 receptor (eticlopride, 60 μM) inhibited its activation (dopamine+etic+SCH: 0.81 ± 0.03, *p* < 0.01 vs. dopamine, *p* < 0.05 vs. control, *n* = 6; Figure S1B). In order to determine which receptor is responsible for ERK1/2 activation, we applied dopamine D1 agonist SKF38393 (20μM, 10 min) with or without its antagonist SCH23390 (40 μM). In both brain areas, SKF38393 increased ERK activity (hippocampus: 1.22 ± 0.03, *p* < 0.01*, n* = 8 and insular cortex: 1.30 ± 0.05, *p* < 0.01, *n* = 8; Figures S1C,E), and co-application with SCH23390 inhibited this activation in both brain areas (hippocampus: 1.05 ± 0.03, *p* < 0.01 vs. SKF38393, *n* = 8, and insular cortex: 1.12 ± 0.06, *p* < 0.01 vs. SKF38393, *n* = 8; Figures S1C,E). However, application of the D2 agonist quinpirole (20 μM, 10 min) did not produce any effect on ERK1/2 in either the hippocampus (1 ± 0.04, *p* = 0.09, *n* = 8; Figure S1C) or insular cortex (1.04 ± 0.06, *p* = 0.515). Moreover, co-application of the D2 agonist quinpirole with D1 antagonist SCH23390 did not result in ERK1/2 activation in either the hippocampus (Figure S1D) or insular cortex (Figure S1F). These results suggest that D1 but not D2 receptors activate ERK1/2 in the mature hippocampus and insular cortex.

### ACTIVATION OF ERK1/2 BUT NOT NR2B Y1472 BY DOPAMINE IS NMDA RECEPTOR-DEPENDENT

**FIGURE 1:**
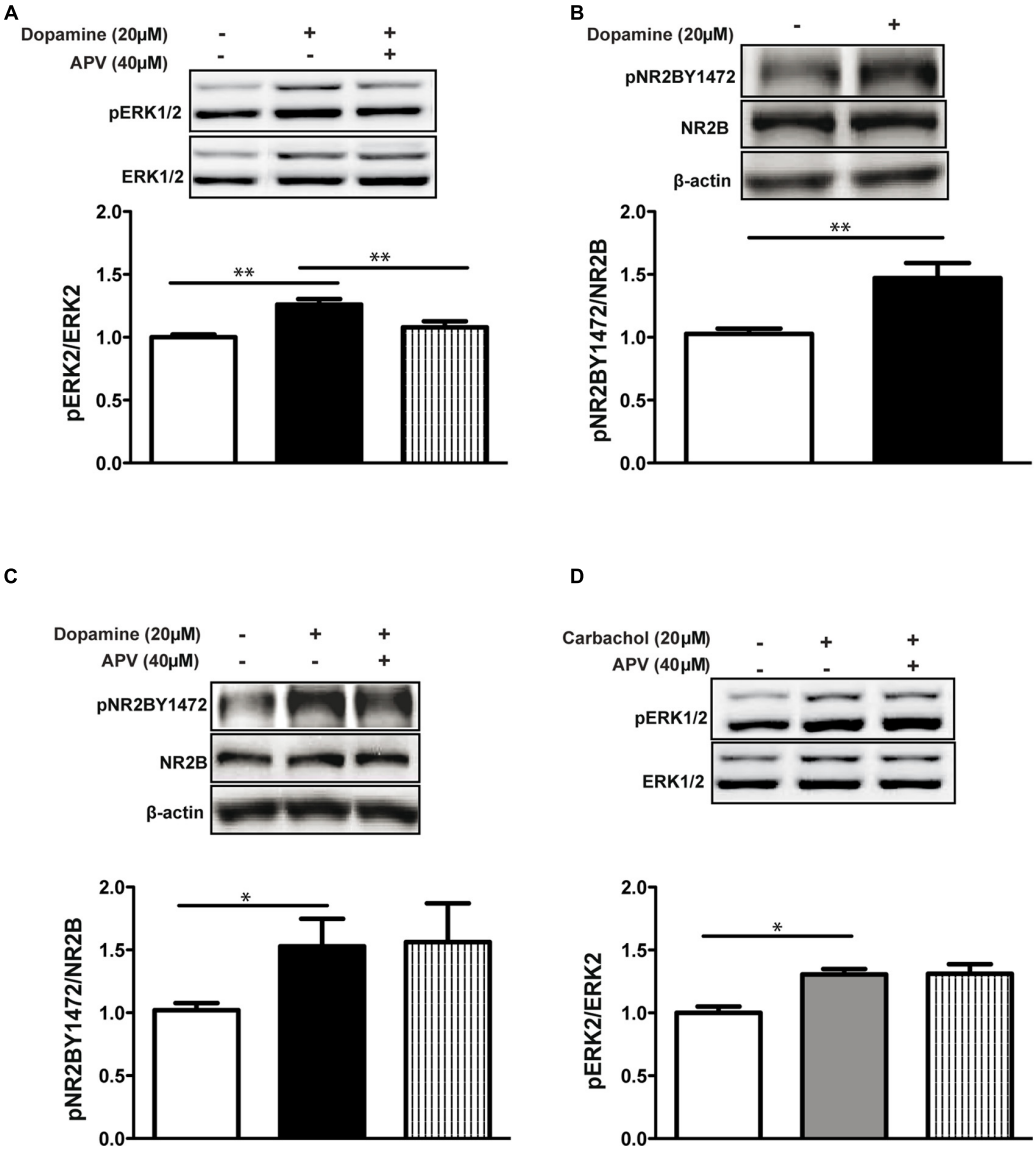
**Activation of ERK1/2 but not NR2B Y1472 by dopamine is NMDA receptor-dependent. (A)** Dopamine (20 μM, 10 min) increases phosphorylation of ERK1/2 in rat hippocampal slices. NMDA receptor antagonist APV (40 μM) abolishes this effect (**p < 0.01, *n* = 6). **(B)** Dopamine (20 μM, 10 min) increases NR2B Y1472 phosphorylation (**p < 0.01, *n* = 24). **(C)** APV (40 μM) does not affect NR2B Y1472 phosphorylation (*p < 0.05, *n* = 12). **(D)** Carbachol (20 μM, 10 min) increases ERK1/2 activation (*p < 0.05, *n* = 12) independently of the NMDA receptor. Data are means ± SEM.

Similarly to Kaphzan et al. (2006), we show that co-application of dopamine with the NMDA receptor antagonist APV (D(-)-APV (D-2-amino-5-phosphonovaleric acid, 40 μM) inhibited ERK1/2 activation in hippocampal slices (dop+APV: 1.07 ± 0.04, *p* < 0.01 vs. dop, *p* < 0.01 dop vs. control, *n* = 6; **Figure 1A**). These results indicate that indeed the convergence of the dopaminergic and glutamatergic inputs converge on the NMDA receptor. Following the results above (**Figure 1A**), we investigated whether the convergence of the dopamine and glutamate signals on the NMDA receptor is mediated via the phosphorylation of the NR2B Y1472 residue. Indeed, dopamine application increased NR2B Y1472 phosphorylation compared to control in hippocampal slices (1.47 ± 0.12, *p* < 0.01, *n* = 24; **Figure 1B**). We next examined whether this NR2B Y1472 phosphorylation was NMDA receptor-dependent. Interestingly, blocking the NMDA receptor with 40 μM APV did not affect NR2B Y1472 phosphorylation induced by dopamine (dop+APV: 1.56 ± 0.2, *p* = 0.92 vs. dop, *n* = 12; **Figure 1C**), indicating that NR2B Y1472 phosphorylation is NMDA receptor independent.

### DOPAMINE BUT NOT ACETYLCHOLINE ACTIVATES ERK1/2 SPECIFICALLY VIA THE NMDA RECEPTOR

As known from previous studies, other neuromodulators such as acetylcholine can increase ERK1/2 activation in various brain areas (Rosenblum et al., 2000). In order to test if ERK1/2 activation via the NMDA receptor is mAChR dependent, we treated slices with the agonist of the muscarinic receptors, carbachol (Cch, 20 μM). Similarly to dopamine, carbachol leads to ERK1/2 activation (1.30 ± 0.02, *p* < 0.05, *n* = 12; **Figure 1D**). However, application of APV did not inhibit Cch-dependent ERK1/2 (Cch+APV: 1.31 ± 0.04, *p* = 0.96 vs. Cch, *n* = 12; **Figure 1D**). These results suggest segregation between dopamine and acetylcholine effects in the mature brain.

### DOPAMINE D1 BUT NOT D2 RECEPTOR INDUCES NR2B Y1472 PHOSPHORYLATION IN THE HIPPOCAMPUS AND INSULAR CORTEX

Following our observation that dopamine induces ERK1/2 activation and NR2B Y1472 phosphorylation, we tested whether NR2B Y1472 phosphorylation was also D1 receptor-selective. We treated hippocampal and insular cortex slices with D1 or D2 agonists separately or concomitantly with their antagonists. Immunoblot analysis showed that indeed the D1 but not D2 receptor agonists mediate NR2B Y1472 phosphorylation in both hippocampus and insular cortex (hippocampus: 1.20 ± 0.05, *p* < 0.05, *n* = 9, insular cortex: 1.28 ± 0.06, *p* < 0.05, *n* = 9; Figures S2A,C), and that co-application of SKF38393 with the D1 receptor antagonist SCH23390 abolished this effect (hippocampus: 1.05 ± 0.07, *p* < 0.05 vs. SKF38393, *n* = 9; insular cortex: 1.04 ± 0.09, *p* < 0.05 vs. SKF38393; Figures S2A,C). Similarly to the results with ERK1/2 (Figure S1), the D2 agonist quinpirole had no effect on NR2B Y1472 phosphorylation in these areas. Moreover, co-application of the D1 antagonist SCH23390 had no effect on NR2B Y1472 phosphorylation (Figures S2B,D). These results demonstrate that dopamine D1 receptor induces both ERK1/2 activation and NR2B Y1472 phosphorylation in mature hippocampus and insular cortex.

### ERK1/2 AND NR2B Y1472 PHOSPHORYLATION IS Src FAMILY KINASE-DEPENDENT

**FIGURE 2:**
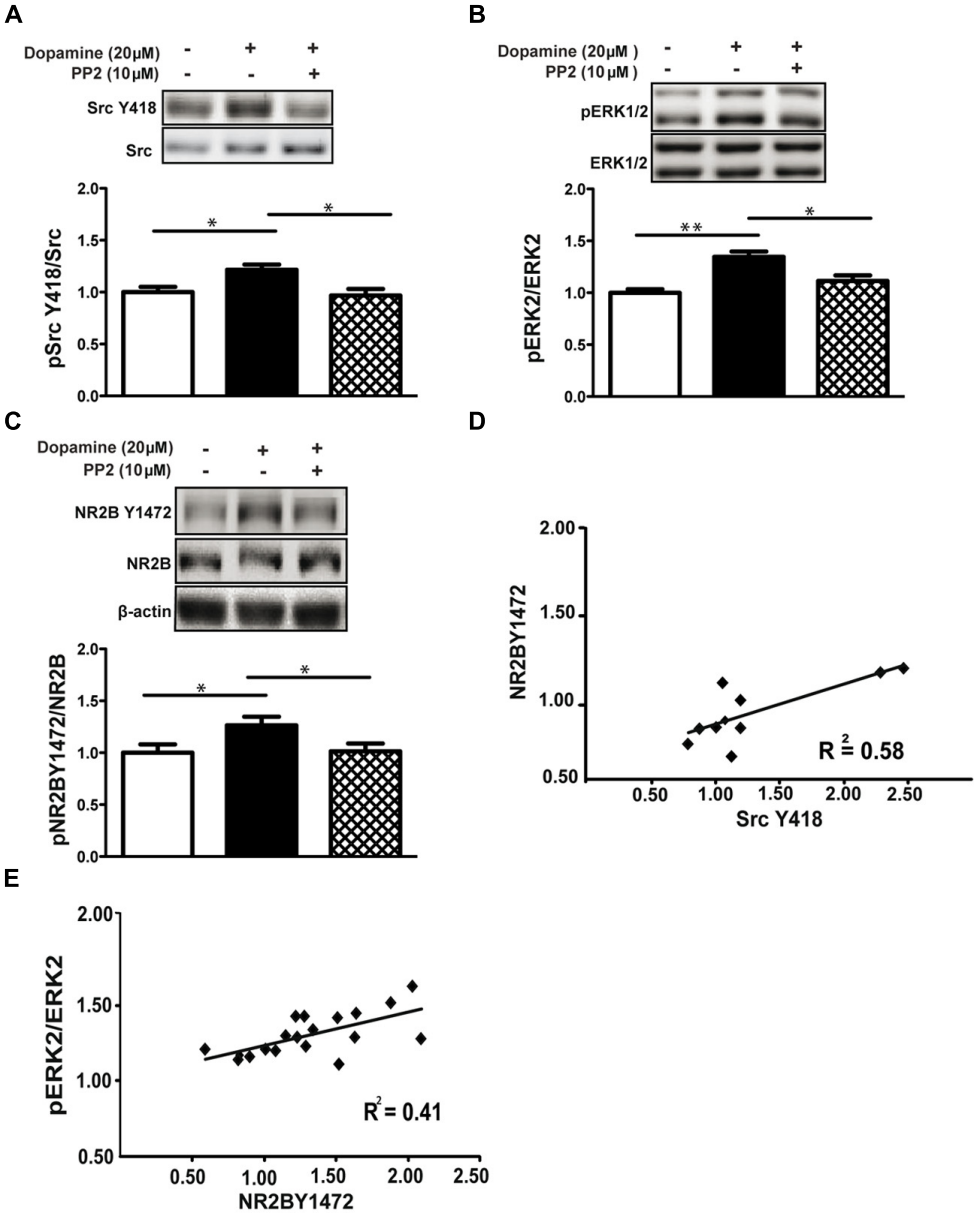
**ERK1/2 and NR2B Y1472 phosphorylation by dopamine is Src family kinase-dependent. (A)** Dopamine (20 μM, 10 min) increases Tyr-418-Src family kinases (pSFKs) phosphorylation in rat hippocampal slices (*p < 0.05), and the SFK inhibitor, PP2 (10 μM), blocks this effect (*p < 0.05 vs. dopamine, *n* = 16). **(B)** Dopamine induces phosphorylation of ERK1/2 (**p < 0.01). This induction is blocked by PP2 (*n* = 16, *p < 0.05 vs. dopamine). **(C)** PP2 blocks the dopamine-induced NR2B Y1472 phosphorylation (*n* = 16, *p < 0.05 vs. control and *p < 0.05 vs. dopamine). **(D)** Positive correlation between NR2B Y1472 and Src Y418 phosphorylation following dopamine (*r* Pearson = 0.58, *p < 0.05). **(E)** Dopamine-induced Y1472 NR2B phosphorylation is correlated with the pERK levels (*r* Pearson = 0.41,*p < 0.05). Data are means ± SEM.

Src family kinases (SFKs) modulate the NMDA receptor function and are important for different aspects of the receptor properties including for trafficking, mainly through phosphorylation of the NR2B subunit at Y1472 (Dunah and Standaert, 2001; Dunah et al., 2004). We therefore examined the role of SFKs in NMDA receptor-dependent ERK1/2 activation induced by dopamine. Dopamine induced SFK phosphorylation of ERK1/2 at Y418 (1.22 ± 0.04, *p* < 0.05, *n* = 10) while the SFK inhibitor PP2 (10 μM) blocked phosphorylation on Y418 (0.96 ± 0.06, *p* < 0.05 vs. dop, *n* = 16; **Figure 2A**). Moreover, dopamine increased phosphorylation of ERK1/2 (1.31 ± 0.05, *p* < 0.01, *n* = 10) and NR2B Y1472 (1.26 ± 0.08, *p* < 0.05, *n* = 16; **Figures 2B,C**). Inhibition of the SFKs with PP2 blocked dopamine-dependent tyrosine phosphorylation of NR2B Y1472 (dop+PP2: 1.09 ± 0.07, *p* < 0.05 vs. dop, *n* = 16; **Figure 2C**) and, more importantly, inhibited ERK1/2-increased activation at the same time (dop+PP2: 1.11 ± 0.05, *p* < 0.05 vs. dop, *n* = 16; **Figures 2B,C**). In addition, we observed correlations between ERK1/2 activation and phosphorylation of NR2B Y1472 (*r* = 0.41; **Figure 2E**), and between SFK activation and NR2B Y1472 phosphorylation (*r* = 0.58, *p* < 0.05; **Figure 2D**).

### DOPAMINE-DEPENDENT NR2B Y1472 PHOSPHORYLATION IS ESSENTIAL FOR ERK1/2 ACTIVATION

**FIGURE 3:**
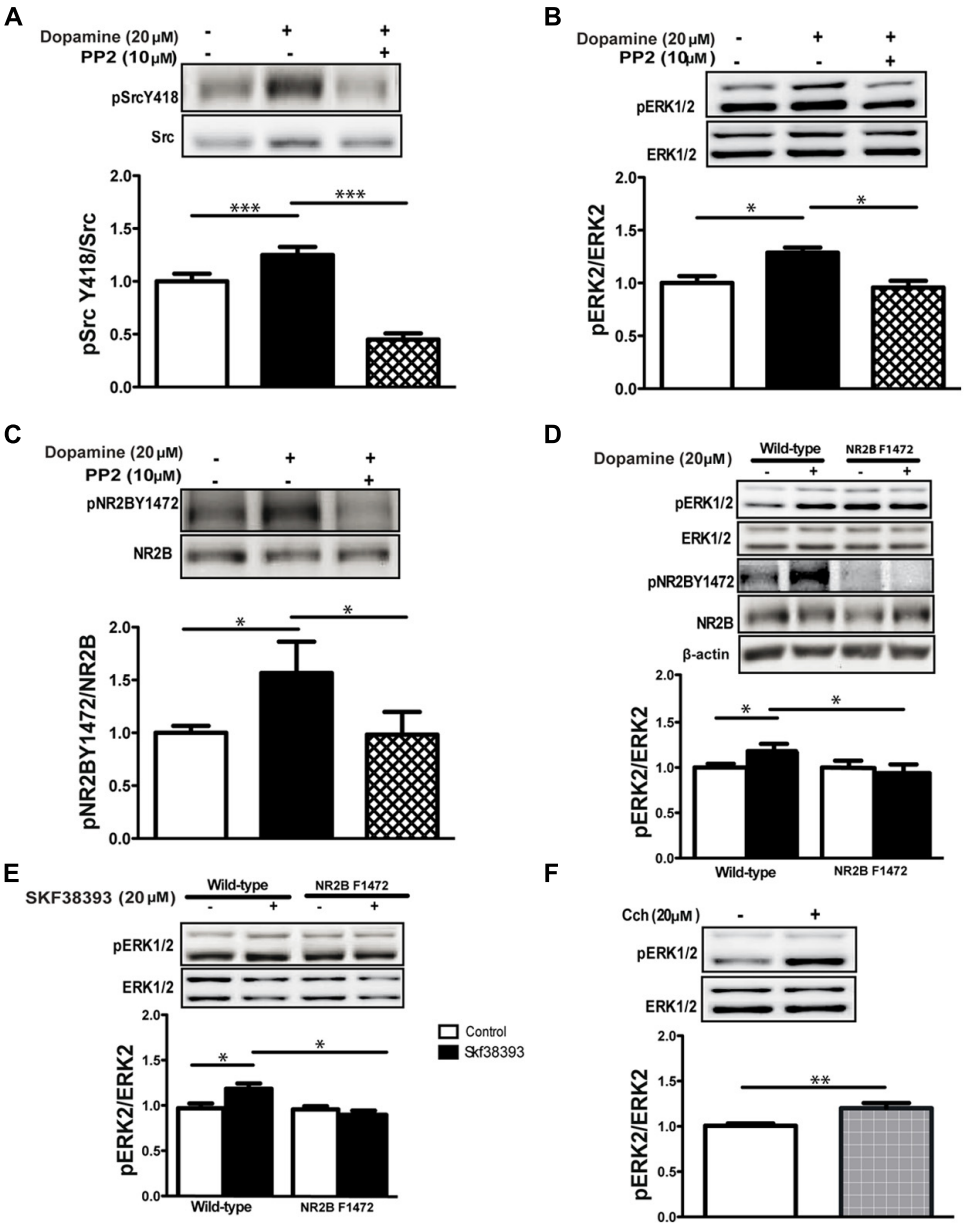
**Dopamine-induced NR2B Y1472 phosphorylation by SFKs is necessary for ERK activation. (A)** Dopamine (20 μM, 10 min) increases phosphorylation levels of Src Y418 (***p < 0.001 vs. control, *n* = 6), **(B)** pERK1/2 (*p < 0.05 vs. control, *n* = 6), **(C)** NR2B Y1472 (*p < 0.05 vs. control, *n* = 6) in C57BL/6 mice hippocampal slices. PP2 (10 μM) prevents these dopamine-induced increases in phosphorylation levels (*n* = 10). **(D)** Dopamine-induced elevation of phosphorylation of ERK1/2 is abolished in hippocampal slices from NR2B F1472 (*p < 0.05 vs. control for wild-type mice, *n* = 6). **(E)** SKF38393 application (20 μM, 10 min) increases the phosphorylation of ERK1/2 in the wild-type mice hippocampal slices but not in the NR2B F1472 mice (*p < 0.05 for wild-type, *n* = 6). **(F)** Carbachol (Cch; 20 μM, 10 min) application increases the phosphorylation of ERK1/2 in NR2B F1472 slices (**p < 0.01, *n* = 12). Data are means ± SEM.

In order to test the general significance of these results to other species and to use transgenic mice to demonstrate causality, we repeated the experiment described above in C57BL/6 wild-type mice. As we had seen in the rat, dopamine increased activation of the SFKs in the hippocampus of mice (1.24 ± 0.06, *p* < 0.001, *n* = 6; **Figure 3A**) and increased phosphorylation of ERK1/2 (1.28 ± 0.04, *p* < 0.05, *n* = 6; **Figure 3B**) and NR2B Y1472 (1.56 ± 0.21, *p* < 0.05, *n* = 6; **Figure 3C**). Co-application with the inhibitor PP2 blocked the activation of SFKs (dop+PP2: 0.44 ± 0.05, *p* < 0.001 vs. dop, *n* = 10; **Figure 3A**) and phosphorylation of ERK1/2 (dop+PP2: 0.95 ± 0.06, *p* < 0.05 vs. dop, *n* = 10) and NR2B Y1472 (dop+PP2: 0.98 ± 0.2, *p* < 0.05 vs. dop, *n* = 10; **Figures 3B,C**). These data suggest that dopamine induces ERK1/2 activation via NR2B Y1472 phosphorylation by the SFKs in mice as in rats.

In light of the above results, we hypothesized that NR2B Y1472 phosphorylation may be upstream and essential for ERK1/2 activation by dopamine. In order to test this hypothesis, we tested hippocampal slices from non-phosphorylatable NR2B F1472 mutant mice and their wild-type littermates (Nakazawa et al., 2006). Two-way ANOVA followed by *post hoc* analysis showed that dopamine induced ERK activation in the wild-type but not NR2B F1472 mice (wild-type: 1.20 ± 0.04, *F* = 4.90, *p* < 0.05; **Figure 3D**). There was a significant effect of the genotype (*F* = 6.01; *p* < 0.05). Moreover, there was a significant interaction between treatment × genotype (*F* = 5.13, *p* < 0.05; **Figure 3D**). Similar results were found with the D1 receptor agonist SKF38393 which activated ERK1/2 in wild-type mice (wild-type: 1.20 ± 0.03, *F* = 3.94, *p* < 0.05; **Figure 3E**). There was a significant effect of the genotype (*F* = 5.28; *p* < 0.05) and also interaction between treatment × genotype (*F* = 5.28, *p* < 0.05; **Figure 3E**). Following our results above that muscarinic receptor-dependent ERK1/2 activation is NMDA receptor independent (i.e., using APV; **Figure 1D**), we hypothesized that carbachol will induce ERK1/2 activation in both wild-type and NR2B F1472 hippocampal slices. Indeed, application of Cch induced ERK1/2 activation in the NR2B F1472 mutant mice similar to that in the wild-type ones (1.20 ± 0.02, *p* < 0.01, *n* = 12; **Figure 3F**). These results indicate the pathway connecting specifically dopamine, NR2B Y1472 phosphorylation, and ERK1/2 activation in the mature brain.

### BOTH NOVEL TASTE- AND DOPAMINE D1 AGONIST-INDUCED ERK1/2 ACTIVATION ARE NR2B Y1472-DEPENDENT

**FIGURE 4:**
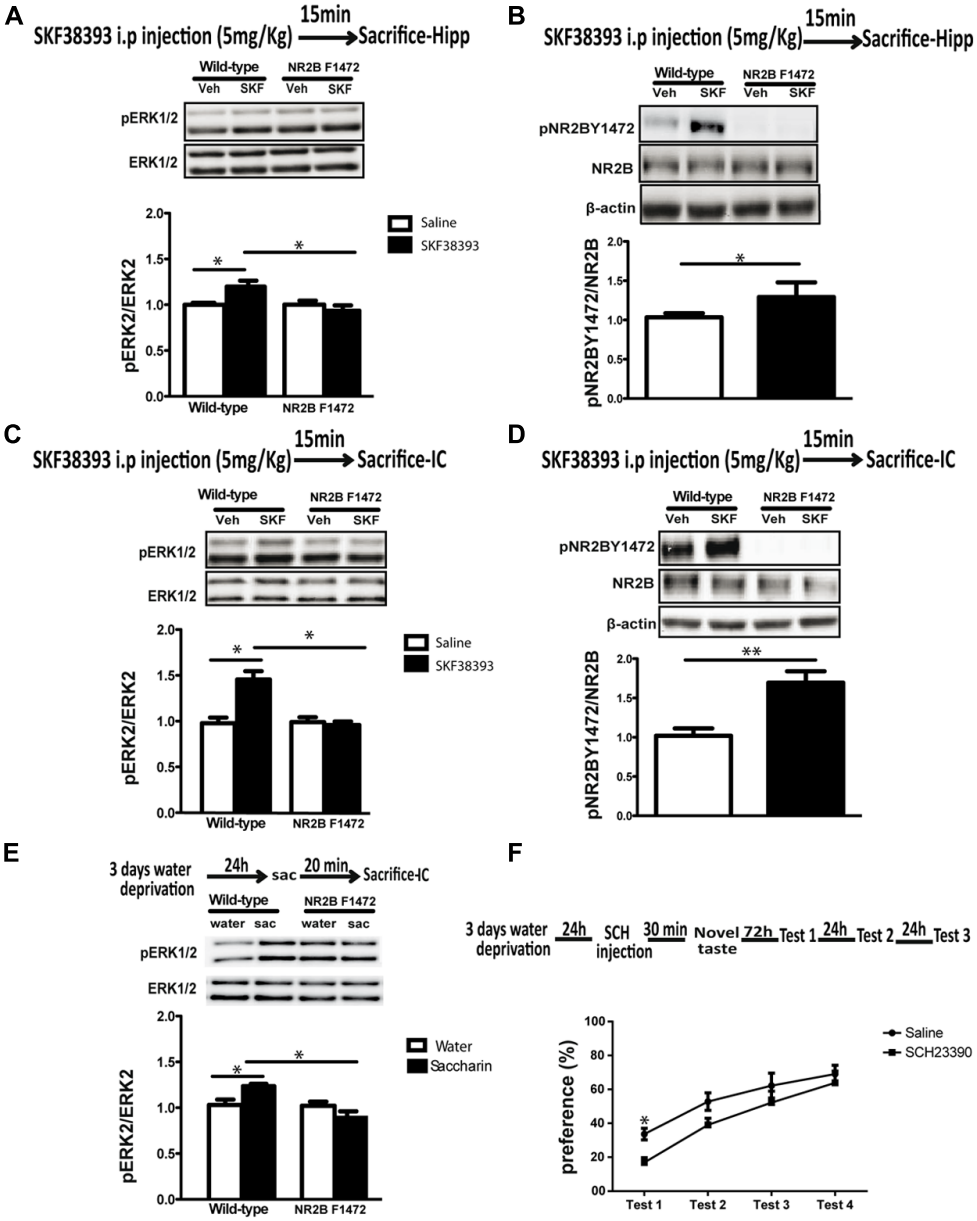
**Dopamine D1 receptor induces ERK1/2 and NR2B Y1472 phosphorylation *in vivo* in both hippocampus and insular cortex. (A)** Injection of SKF38393 (5 mg/kg, i.p.) increases pERK1/2 levels in wild-type mice hippocampus, but not in NR2B F1472 mice (*p < 0.05 vs. control and *p < 0.05 vs. wild-type SKF38393, *n* = 5). **(B)** Injection of SKF38393 increases NR2B Y1472 phosphorylation levels in the wild-type mice hippocampus (*p < 0.05 vs. control), but not in NR2B F1472 mice (*n* = 6). **(C)** SKF38393 (5 mg/kg, i.p.) increases pERK1/2 and **(D)** pNR2B Y1472 levels in the insular cortex wild-type mice but not in the NR2B F1472 (pERK1/2: *p < 0.05 vs. control and *p < 0.05 vs. wild-type SKF38393, *n* = 5; pNR2B Y1472L: **p < 0.01 vs. control, *n* = 6). **(E)** Novel taste exposure for 20 min induces the phosphorylation of ERK1/2 in wild-type mice but not in NR2B F1472 mice (*p < 0.05 vs. water and *p < 0.05 vs. wild-type saccharin, *n* = 10). **(F)** Injection of D1 receptor antagonist SCH23390 (0.05 mg/kg, i.p.) prior to novel taste exposure impairs taste memory 72 h after in the first retrieval day (*p ≤ 0.05, *n* = 9). Data are means ± SEM.

In order to evaluate the physiological effect of the pathway described above *in vivo* and its impact on cortical-dependent novelty learning NR2B F1472 mutant mice and wild-type littermates were injected with either saline or low concentration of SKF38393 (5 mg/kg) and both hippocampus and insular cortex tissues were collected 15 min post-injection. Western blotting analysis showed that indeed *in vivo* as *in vitro*, D1 receptor stimulation induces NR2B Y1472 phosphorylation in wild-type hippocampus (1.25 ± 0.10, *p* < 0.05, *n* = 6; **Figure 4B**) and insular cortex (1.56 ± 0.09, *p* < 0.01, *n* = 6; **Figure 4D**). However, two-way ANOVA followed by *post hoc* analysis showed that ERK1/2 activation following the D1 agonist treatment was detected in the wild-type mice but not in the NR2B F1472 mice in both brain areas (hippocampus: 1.22 ± 0.05, *F* = 3.98*, p* < 0.05; insular cortex: 1.49 ± 0.09, *F* = 6.34, *p* < 0.05; **Figure 4A**). There was also a significant effect of the genotype (*F* = 6.91, *p* < 0.05) and interaction of treatment × genotype in the hippocampus (*F* = 3.92, *p* < 0.05); **Figure 4A**) and in the insular cortex (*F* = 5.72, *p* < 0.05) for the genotype (*F* = 5.72, *p* < 0.05; **Figure 4C**). These results demonstrate the necessary role of NR2B Y1472 phosphorylation in ERK1/2 activation by dopamine in the mature brain.

In light of the *in vitro* and *in vivo* pharmacological results, we set to examine whether the dopamine-induced NR2B Y1472 phosphorylation was important for consolidation of novel sensory information. It is known that novel taste learning induces both ERK1/2 and NR2B Y1472 phosphorylation in the gustatory cortex which resides within the insular cortex (Belelovsky et al., 2005; Barki-Harrington et al., 2009). Therefore, we hypothesized that impairment of dopamine-dependent ERK1/2 activation in the NR2B F1472 mutant mice would lead to a behavioral deficit in learning of cortical-dependent novel taste information. Wild-type and NR2B F1472 mutant mice were exposed to novel taste (saccharin) and were sacrificed 20 min later for biochemical analysis. Two-way ANOVA followed by *post hoc* analysis revealed that similarly to the D1 agonist, novel taste increased ERK1/2 activation in the wild-type mice, while no ERK1/2 activation was found in the NR2B F1472 mice (wild-type: 1.20 ± 0.04, *F* = 3.82, *p* < 0.05; **Figure 4E**). There was also significant effect of the genotype (*F* = 9.51, *p* < 0.01) and interaction of treatment × genotype (*F* = 8.30, *p* < 0.01). In addition and as was reported before, novel taste induced NR2B Y1472 phosphorylation in the wild-type but not in the NR2B F1472 mice (data not shown). Moreover, novel taste memory is dopamine D1 receptor dependent as was shown previously (Berman et al., 2000; Cannon et al., 2005), since blocking the D1 receptor with SCH23390 (0.05 mg/kg, i.p.) 30 min prior to the novel taste exposure impaired the memory of the taste in the first choice test day in wild-type mice (33 ± 0.03% for saline-injected mice vs. 16 ± 0.08% for SCH-injected mice, *p* < 0.05, *n* = 9). There was significant effect of the testing days, indicating normal attenuation of neophobia (**Figure 4F**). There were no differences in the total drinking volume during the test days (Table S1).

### NR2B Y1472 PHOSPHORYLATION IS ESSENTIAL FOR NOVEL TASTE LEARNING

**FIGURE 5:**
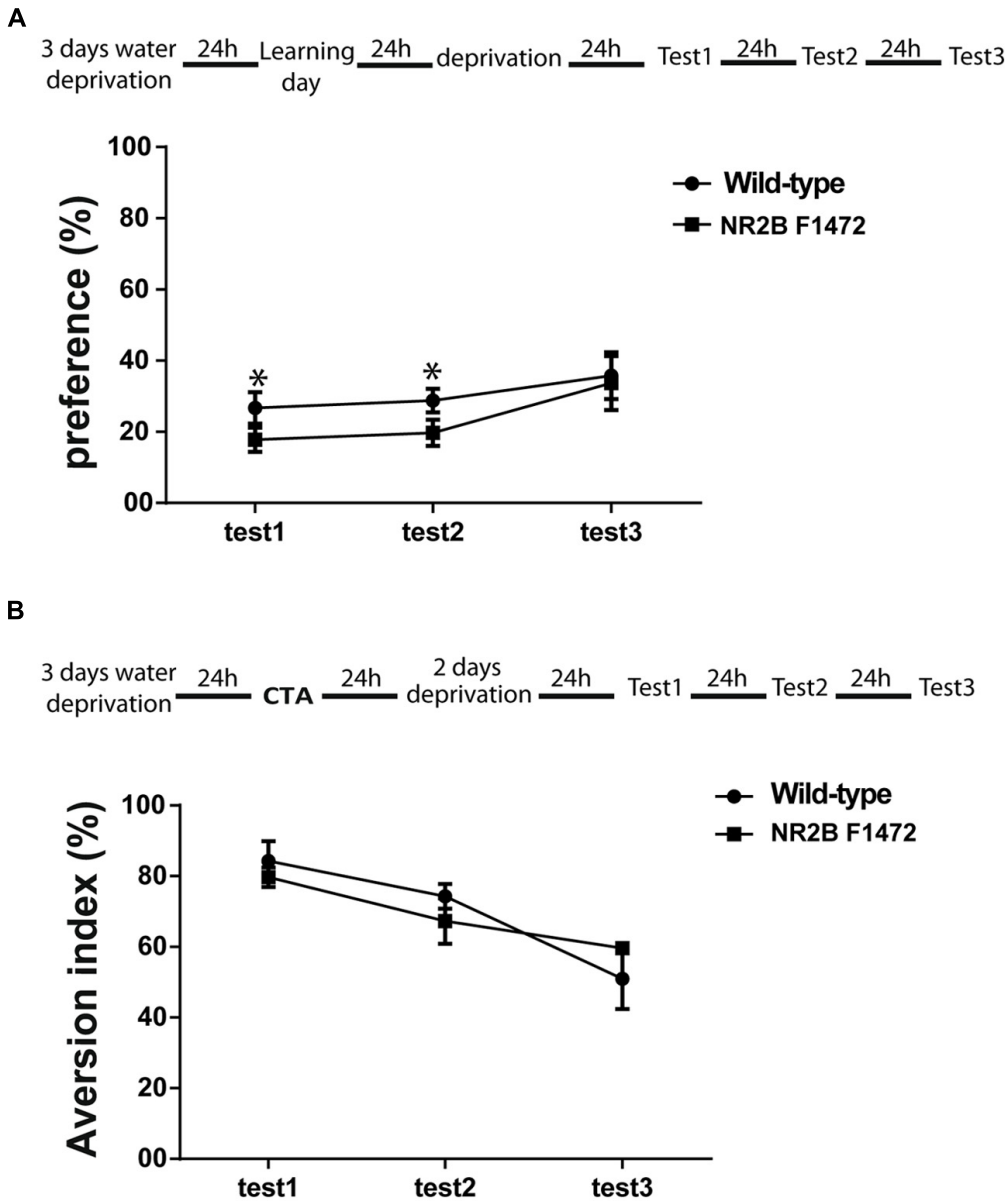
**NR2B Y1472 phosphorylation is necessary for novel taste learning. (A)** NR2B F1472 mice prefer significantly less novel taste on the first and second choice tests (*p < 0.05). However, attenuation of neophobia is normal as can be seen by the slope (wild-type *n* = 8, NR2B F1472 *n* = 9). **(B)** Conditioned taste aversion memory was normal in the NR2B F1472 mice compared to their wild-type (*n* = 9). Data are means ± SEM.

The results above suggested that dopamine plays a key role in novel taste memory. We thus hypothesized that impairment of dopamine-dependent ERK1/2 activation in the NR2B F1472 mutant mice would lead to a behavioral deficit in learning of novel taste information. C57BL/6 wild-type and NR2B F1472 mutant mice were subjected to two cortical-dependent learning tasks: attenuation of neophobia of novel taste learning and CTA. NR2B F1472 mutant mice showed lower preference to the novel taste saccharin than the wild-type mice at the first test day (26 ± 0.04% for wild-type mice vs. 17 ± 0.03% for NR2B F1472 mice, *n* = 16) and second test day (28 ± 0.03% for the wild-type mice vs. 19 ± 0.03% for NR2B F1472 mice, *n* = 16; **Figure 5A**).

Repeated-measures ANOVA showed that there was a significant difference between the groups (*F* = 641, *p* < 0.001) and also a significant effect of the test days (*F* = 6.34, *p* < 0.05; **Figure 5A**). There were no differences in total volume drinking during the test days (Table S2). Associative memory was normal in the NR2B F1472 mice (**Figure 5B**). The NR2B F1472 mice showed high aversion index at the first test day similar to the wild-type littermates (84.25 ± 0.11 for wild-type mice and 80.21 ± 0.06 for the NR2B F1472 mice, *n* = 9, *p* = 0.45). Repeated-measures ANOVA showed a significant effect of the testing days (*F* = 3.51, *p* < 0.05; **Figure 5B**) indicating normal extinction for both groups. There were no differences in the total drinking volume during the test days (Table S3). These results imply of the importance of the NR2B Y1472 in learning of novel taste information.

## DISCUSSION

Our study describes a molecular pathway of dopamine and glutamate signaling converging on the NMDA receptor itself, which results in ERK1/2 activation in the mature hippocampus and cortex. Dopamine D1 receptor but not D2 induces ERK1/2 activation in an NMDA receptor-dependent manner. Specifically, D1 receptor activation leads via the SFKs to phosphorylation of NR2B at residue Y1472, which in turn, activates ERK1/2. Furthermore, we have found a correlation between NR2B Y1472 phosphorylation and ERK1/2 activation in the mature brain. Using NR2B F1472 mutant mice, we have demonstrated *in vitro* and *in vivo* the necessity of the NR2B Y1472 for ERK1/2 activation following D1 receptor activation and for novel taste learning.

Our results indicate that it is specifically the D1 receptor that is responsible for ERK1/2 activation in both mature hippocampus and insular cortex (Figure S1), as activation of the D2 receptor while blocking the D1 receptor leads to no such effect. This is in agreement with previous studies which have shown both the necessity of D1 receptor (and not D2) in a prefrontal cortex-dependent, long-term memory, and that D1 receptor can affect long-term recognition memory via ERK1/2 (Belelovsky et al., 2005; Nagai et al., 2007).

A large body of evidence supports the pivotal role of the dopamine–NMDA receptor interactions in various learning and memory tasks. D1 receptor and NMDA receptor interactions are required for ERK1/2 activation following exposure to a novel environment (Sarantis et al., 2011), attention control (Agnoli and Carli, 2011), novel information (Coccurello et al., 2012), and long-term memory of taste learning (Guzman-Ramos and Bermudez-Rattoni, 2011) as well as addiction (Zhang et al., 2004; Jenab et al., 2005). Stimulation of both dopamine D1 and NMDA receptors in the striatum also activates ERK1/2 (Valjent et al., 2005). In accordance with these findings, we report here that dopamine D1 and NMDA receptors are also required for ERK1/2 activation in both mature hippocampus and cortex, and that dopamine-dependent ERK1/2 activation is NMDA receptor-dependent, since blocking the NMDA receptor with APV prevented the dopamine-dependent ERK1/2 activation.

In spite of the abovementioned evidence for dopamine–NMDA receptor interplay, little is known about the mechanism underlying this interaction. Previous studies have suggested different mechanisms for dopamine–NMDA interactions, whether via direct protein–protein interactions or via G protein-dependent/-independent regulation of the NMDA receptor (Lee et al., 2002; Lee and Liu, 2004; Martina and Bergeron, 2008). In this study, we report that dopamine D1 receptor but not D2 induces the phosphorylation of the NR2B subunit at the Y1472 residue both in the hippocampus (shown *ex vivo*) and the insular cortex (shown *in vivo*). This mechanism was found also in other brain areas. For example, in striatal and pre-frontal cortex neurons, activation of the D1 receptors increases tyrosine phosphorylation of NR2A and NR2B, and produces rapid alteration in the distribution of the NR1, NR2A, and NR2B subunits of the NMDA receptor, with accumulation of these proteins in synaptosomal membranes (Dunah and Standaert, 2001). Furthermore, activation of dopamine D1 receptor leads to trafficking of the NMDA receptor in the striatum in a Fyn protein tyrosine kinase-dependent manner (Dunah et al., 2004; Hallett et al., 2006), and recently it was shown in the striatum that cocaine can induce NR2B Y1472 phosphorylation via the D1 receptor (Pascoli et al., 2011). However, our data show that the dopamine-induced NR2B Y1472 phosphorylation was not NMDA receptor-dependent, suggesting a metabotropic function of the NMDA receptor as was shown to be important for long-term depression (Kessels et al., 2013; Nabavi et al., 2013).

NMDA receptor-dependent induction of ERK1/2 signaling is important for NMDA receptor-dependent LTP/LTD in the CA1 region of the hippocampus (Kanterewicz et al., 2000; Thiels et al., 2002) and for transcription of early genes in cortical neurons and the hippocampus (Xia et al., 1996; Chung et al., 2000). The NR2A and NR2B subunits can differentially modulate ERK1/2 activation: NR2A promotes surface expression of GluR1 and NR2B inhibits its expression via inhibition of ERK1/2 signaling (Kim et al., 2005).

Because our results show that dopamine leads to phosphorylation of the NMDA receptor at NR2B Y1472 in both rats and mice, we further examined whether NR2B Y1472 phosphorylation is necessary for ERK1/2 activation. Using NR2B F1472 mice which do not have NR2B Y1472 phosphorylation, we have shown that neither application of dopamine nor the D1 agonist SKF38393 to hippocampal slices, nor i.p. injection of dopamine results in ERK1/2 activation in the hippocampus and cortex of these mice, unlike their wild-type littermates. These results provide evidence for the importance of the convergence of the dopamine and glutamate input on the NMDA receptor in order to activate ERK1/2.

According to our data, blocking the SFKs reduced NR2B Y1472 phosphorylation following dopamine application. More importantly, blocking the SFKs inhibited ERK1/2 activation in both rats and mice, suggesting that normal function of SFKs is necessary for ERK1/2 activation in the mature hippocampus and cortex, as seen before in the striatum (Pascoli et al., 2011). This is in line with a previous study, which showed that dopamine-induced PKA and SFK activation as well as NR2B-containing NMDA receptors are crucial for LTP enhancement (Stramiello and Wagner, 2008).

There are many reports in the literature regarding the necessity of dopaminergic receptors and the NMDA receptor to process information concerning novel stimuli involving different brain regions. For example, a recent study showed that dopamine D1 receptor and NMDA receptor interactions were required for ERK1/2 activation following exposure to a novel environment (Sarantis et al., 2011). In addition, previous studies have shown that novel stimuli increase dopamine release in the hippocampus and prefrontal cortex (Ljungberg et al., 1992; Ihalainen et al., 1999; Li et al., 2003), and that novelty-seeking behavior is reduced following elimination of dopamine transmission, especially in the nucleus accumbens (Hooks and Kalivas, 1995). Exposure of rats to novel environments promoted LTP in a dopamine D1/D5-dependent manner (Li et al., 2003). The NMDA receptor NR2B subunit was shown to be important in various learning and memory tasks, such as fear conditioning (Nakazawa et al., 2006; Sotres-Bayon et al., 2007; Walker and Davis, 2008; Zhang et al., 2008), conditioned-taste learning, and novel taste learning (Rosenblum et al., 1997; Barki-Harrington et al., 2009). Moreover, deletion of the NR2B subunit from adult granule cells in the dentate gyrus causes a deficit in novelty exploration (Kheirbek et al., 2012).

Studies about taste learning have shown that exposure to novel taste induces NR2B Y1472 phosphorylation (Barki-Harrington et al., 2009) and induces increased pERK levels 20 min following exposure to saccharin as a novel taste (Belelovsky et al., 2005; Elkobi et al., 2008). Moreover, the dopaminergic system, especially the D1/D5 receptor, is necessary for incidental taste learning and CTA. Mice lacking the D1 receptor show impaired CTA for sucrose but not salt (Cannon et al., 2005), and blocking the D1 receptor with SCH23390 in the insular cortex also impairs this learning (Berman et al., 2000). It was also shown that following exposure to novel taste, there is an off-line release of dopamine and glutamate in the insular cortex (Guzman-Ramos et al., 2010). Both D1 and D2 receptors play a role in the fructose-conditioning flavor preference in the amygdala and nucleus accumbens (Fenu et al., 2001; Baker et al., 2003; Bernal et al., 2008) and in glucose-conditioned flavor preference (Touzani et al., 2010). The circuit and neuronal type which express the increased pERK are not known. However, Arc/Arg3.1, whose expression is considered to be pERK-dependent, demonstrates lateralized expression following novel taste learning (Inberg et al., 2013).

Following the pharmacological results and the studies mentioned above, we hypothesized that NR2B F1472 mice would be impaired in novel taste learning compared with wild-type mice. Indeed, we found that NR2B F1472 mice were impaired in attenuation of neophobia to saccharin (novel taste learning) but not in associative learning of CTA. Although our results indicate impaired cortical-dependent learning of the NR2B F1472 mice, it was shown in previous studies that these mice are not impaired in hippocampal-dependent learning (Nakazawa et al., 2006), suggesting a different molecular mechanism for ERK1/2 activation. The importance of NR2B Y1472 phosphorylation following dopamine stimulation for the activation of ERK1/2 and learning of novel information may be due to 1) NR2B Y1472 phosphorylation results in increase of calcium influx to the cell, which leads to more ERK1/2 activation as previously shown (Pascoli et al., 2011), or 2) D1 receptors induce NR2B Y1472 phosphorylation, and increase synaptic expression of NMDA receptors as previously suggested (Dunah et al., 2004). Further work is needed in order to determine which pathway is the one activated in the hippocampus and cortex.

In addition to dopaminergic and glutamatergic signaling, muscarinic transmission also plays an important role in ERK1/2 activation and consolidation of memory, as well as synaptic plasticity. For example, the muscarinic agonist carbachol (Cch) induces prolonged dose-dependent activation of ERK1/2 in the CA1 pyramidal neurons of mice (Rosenblum et al., 2000; Berkeley et al., 2001). In addition, both muscarinic acetylcholine receptors (mAChRs) and ERK1/2 have previously been shown to play a role in LTP and other forms of synaptic modulation (Watabe et al., 2000). In line with these reports, in the present study carbachol (Cch) induced ERK1/2 activation in hippocampal slices. Based on the results that show that D1-induced ERK1/2 activation is NMDA receptor-dependent, we further examined whether mAChRs use similar molecular pathways to activate ERK1/2. We found that ERK1/2 activation by mAChRs was NMDA receptor-independent. Moreover, we found that ERK1/2 was activated in the NR2B F1472 mice following Cch administration, but not following dopamine stimulation. This segregation between dopamine and mAChRs may imply that there are distinct mechanisms, both of which are necessary for induction of ERK1/2 and long-term memory. Whereas dopamine receptors require glutamatergic transmission in order to enhance and maintain LTP in the hippocampus (Frey et al., 1990, 1991; Navakkode et al., 2010), the mAChRs may induce ERK1/2 activation by cAMP and PKC (Kim et al., 1999; Rosenblum et al., 2000; Budd et al., 2001), and not via the NMDA receptor.

In conclusion, we have demonstrated that dopamine converges on the NMDA receptor and leads to phosphorylation of NR2B Y1472 which is both correlated with and necessary for ERK1/2 activation in the mature hippocampus and cortex. Malfunction of dopamine or NMDA receptors causes various types of mental disorders, such as schizophrenia. Our results, taken together with recent findings in schizophrenia patients (Kantrowitz and Javitt, 2010; Weickert et al., 2012), may imply that impairment in dopamine-induced NR2B Y1472 phosphorylation and ERK1/2 activation is involved in schizophrenia-like behavior and thus can function as targets for possible therapy.

## Conflict of Interest Statement

The authors declare that the research was conducted in the absence of any commercial or financial relationships that could be construed as a potential conflict of interest.
