# Supplementary material for: Dopamine-induced tyrosine phosphorylation of NR2B (Tyr1472) is essential for ERK1/2 activation and processing of novel taste information
Source: Front Mol Neurosci. 2014 Jul 18;7:66. doi: 10.3389/fnmol.2014.00066 (PMC4103512; doi:10.3389/fnmol.2014.00066)
Supplement: Supplementary file 1 [file Data_Sheet_1.ZIP › Data_Sheet_3.PDF]

		Acquisition	Test 1	Test 2	Test 3	Test 4
saline	Mean (ml)	1.4	2.2	2	2.1	2.3
	SD	0.33	0.59	0.39	0.32	0.45
SCH	Mean (ml)	1.4	1.9	1.9	2.3	1.9
	SD	0.43	0.47	0.27	0.49	0.40
