# Supplementary material for: Dopamine-induced tyrosine phosphorylation of NR2B (Tyr1472) is essential for ERK1/2 activation and processing of novel taste information
Source: Front Mol Neurosci. 2014 Jul 18;7:66. doi: 10.3389/fnmol.2014.00066 (PMC4103512; doi:10.3389/fnmol.2014.00066)
Supplement: Supplementary file 1 [file Data_Sheet_1.ZIP › Data_Sheet_4.PDF]

		Acquisition	Test 1	Test 2	Test 3
Wild-type	Mean (ml)	1.9	2	2.1	2
	SD	0.3	0.26	0.27	0.16
NR2B F1472	Mean (ml)	1.9	1.9	2	2
	SD	0.23	0.30	0.44	0.69
