# Supplementary material for: Dopamine-induced tyrosine phosphorylation of NR2B (Tyr1472) is essential for ERK1/2 activation and processing of novel taste information
Source: Front Mol Neurosci. 2014 Jul 18;7:66. doi: 10.3389/fnmol.2014.00066 (PMC4103512; doi:10.3389/fnmol.2014.00066)
Supplement: Supplementary file 1 [file Data_Sheet_1.ZIP › Data_Sheet_5.PDF]

		CTA day	Test 1	Test 2	Test 3
Wild-type	Mean (ml)	1.4	1.3	1.6	1.7
	SD	0.31	0.42	0.14	0.47
NR2B F1472	Mean (ml)	1.5	1.4	1.7	1.7
	SD	0.33	0.27	0.31	0.33
