# Supplementary material for: Dopamine-induced tyrosine phosphorylation of NR2B (Tyr1472) is essential for ERK1/2 activation and processing of novel taste information
Source: Front Mol Neurosci. 2014 Jul 18;7:66. doi: 10.3389/fnmol.2014.00066 (PMC4103512; doi:10.3389/fnmol.2014.00066)
Supplement: Supplementary file 1 [file Data_Sheet_1.ZIP › Data Sheets legends.pdf]

1. Supplementary Material

Figure S1. Dopamine D1 but not D2 receptor activates ERK1/2 in hippocampal and insular cortex slices. (A) Dopamine (20µM, 10 min) increases phosphorylation of ERK1/2 in hippocampal slices (***p<0.001 vs. control, n=24). **(B)** Co-application of dopamine with D1 and D2 antagonists (40µM SCH23390 and 60µM eticlopride, respectively) reduces pERK1/2 levels (**p<0.01 vs. dopamine, *p<0.05 vs. control, n=6). **(C)** Application of the D1 agonist SKF38393 (20µM, 10 min) increases phosphorylation of ERK1/2 in hippocampal slices (**p<0.01 vs. control). This effect is abolished by the D1 antagonist SCH23390 (**p<0.01 vs. SKF, n=8). (D) Application of the D2 agonist quinpirole does not change the phosphorylation of ERK1/2. Co-application of quinpirole and SCH23390 does not affect phosphoryation of ERK1/2 (n=8). **(E)** Application of the D1 agonist SKF38393 (20µM, 10 min) increases phosphorylation of ERK1/2 in insular cortex slices (**p<0.01 vs. Control), and the D1 antagonist SCH23390 reduces this effect (**p<0.01 vs. SKF38393, n=8). **(F)** Application of the D2 agonist quinpirole (20µM, 10 min) or co-application of quinpirole and SCH23390 to insular cortex slices had no effect on the phosphorylation of ERK1/2 p>0.05, n=8). Data are means ± SEM.

Figure S2. Dopamine D1 but not D2 receptor induces NR2B Y1472 phosphoryaltion in both hippocampus and insular cortex slices. (A) D1 agonist SKF38393 (20µM, 10 min) increases NR2B Y1472 phosphorylation in hippocampal slices (*p<0.05, n=9). D1 antagonist SCH23390 (40µM) blocks the SKF38393-induced NR2B Y1472 phosphorylation (*p<0.05 vs. SKF38393, n=9). **(B)** D2 agonist quinpirole (20µM) has no effect on NR2B Y1472 phosphorylation and also, D1 antagonist SCH23390 does not change NR2B Y1472 levels (p=0.095, n=9). **(C)** D1 agonist SKF38393 increases NR2B Y1472 phosphorylation in insular cortex slices (*p<0.05 vs. control), whereas D1 antagonist SCH23390 blocks this phosphorylation (*p<0.05 vs. SKF38393, n=9). **(D)** Quinpirole has no effect on NR2B Y1472 phosphorylation in insular cortex slices and also, quinpirole with D1 antagonist SCH23390 does not change the NR2B Y1472 levels (p=0.437 vs. control, n=9). Data are means ± SEM.

Table S3. Total drinking volume of saline and SCH23390-injected mice during the testing days.

Table S4. Total drinking volume of wild-type and NR2B F1472 mice during the attenuation of neophobia tests.

Table S5. Total drinking volume of wild-type and NR2B F1472 mice during CTA day and testing days.
