# Supplementary figures and images for: Dopamine-induced tyrosine phosphorylation of NR2B (Tyr1472) is essential for ERK1/2 activation and processing of novel taste information
Source: Front Mol Neurosci. 2014 Jul 18;7:66. doi: 10.3389/fnmol.2014.00066 (PMC4103512; doi:10.3389/fnmol.2014.00066)

**A**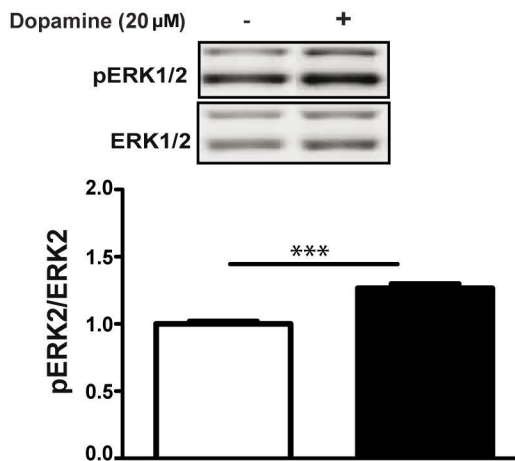**B**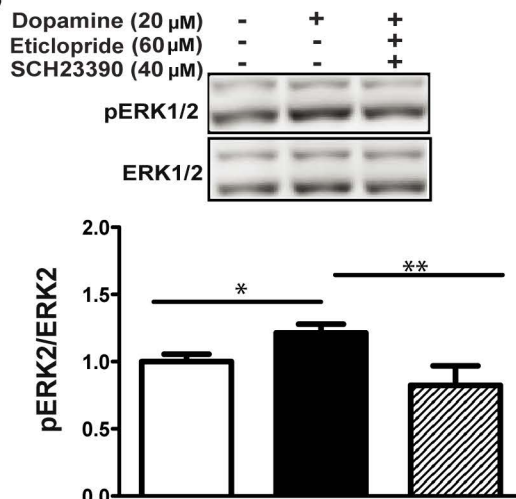**C**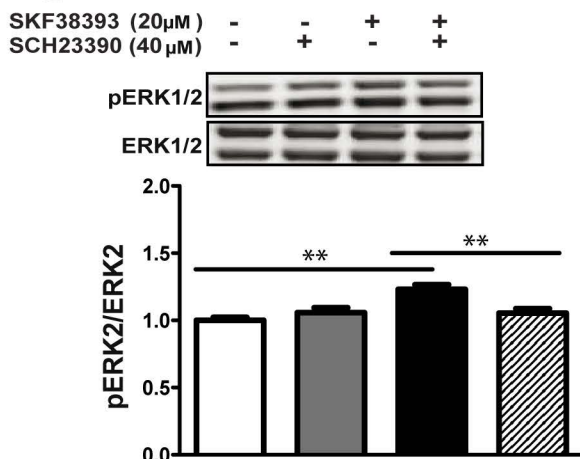**D**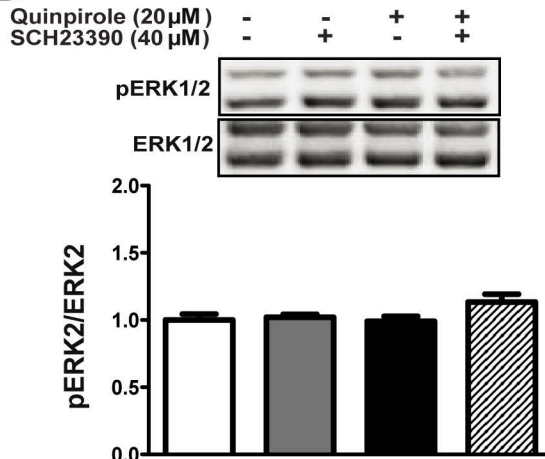**E**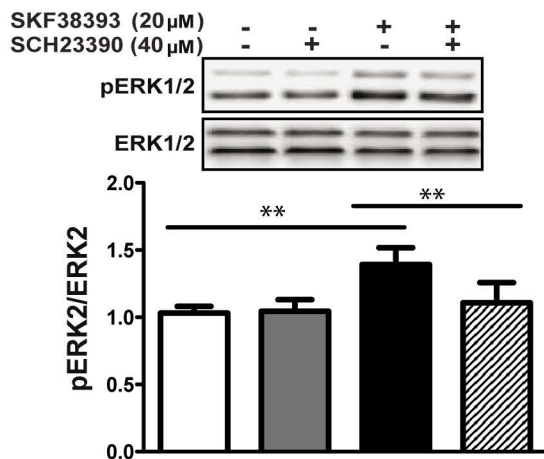**F**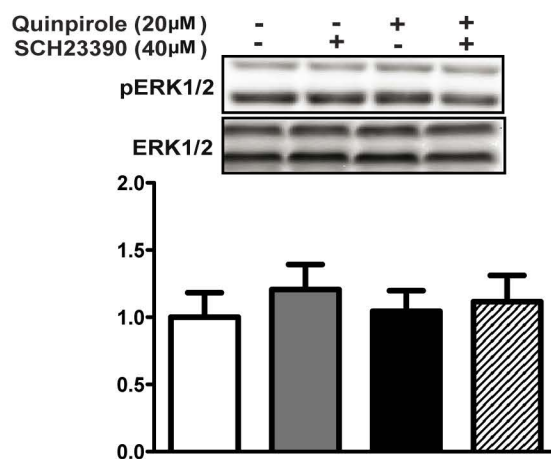

Supplement: Supplementary file 1 [file Data_Sheet_1.ZIP › Data_Sheet_1.PDF]

**A**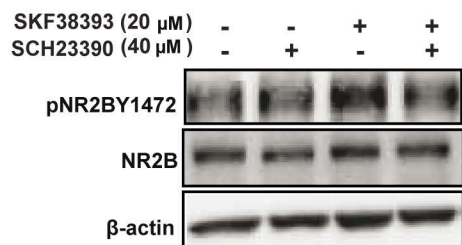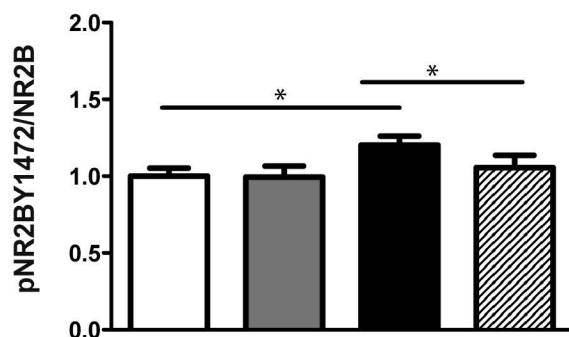**B**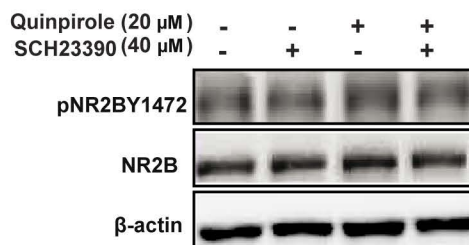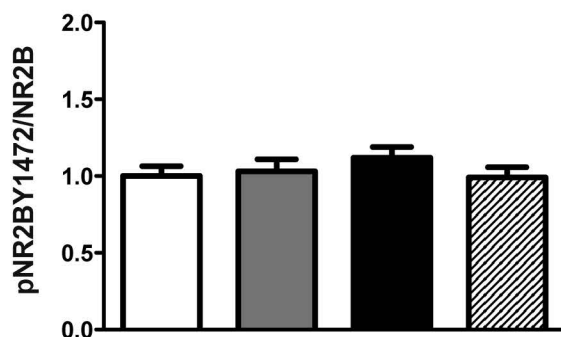**C**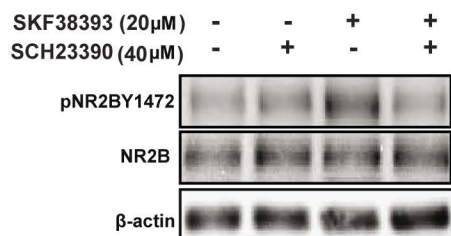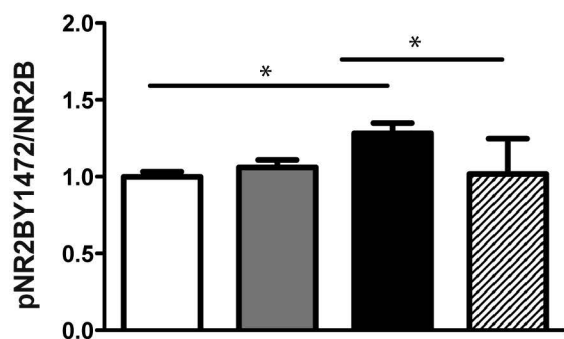**D**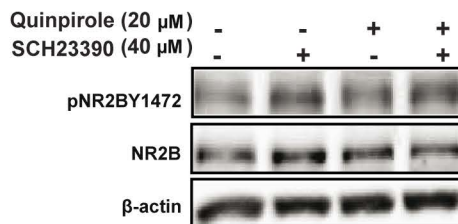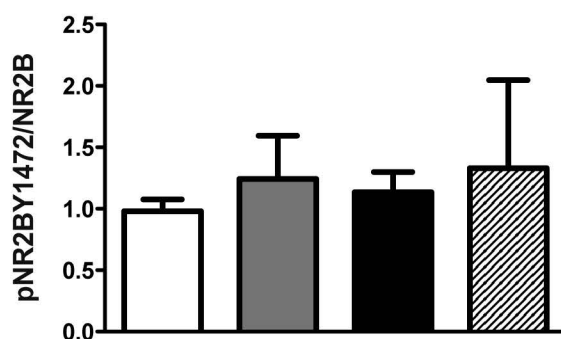

Supplement: Supplementary file 1 [file Data_Sheet_1.ZIP › Data_Sheet_2.PDF]
